# Cellular stress conditions are reflected in the protein and RNA content of endothelial cell-derived exosomes

**DOI:** 10.3402/jev.v1i0.18396

**Published:** 2012-04-16

**Authors:** Olivier G. de Jong, Marianne C. Verhaar, Yong Chen, Pieter Vader, Hendrik Gremmels, George Posthuma, Raymond M. Schiffelers, Marjan Gucek, Bas W.M. van Balkom

**Affiliations:** 1 Department of Nephrology and Hypertension, UMC Utrecht, Utrecht, The Netherlands; 2 National Heart, Lung and Blood Institute, National Institutes of Health, Bethesda, MD, USA; 3 Department of Clinical Chemistry and Hematology, UMC Utrecht, Utrecht, The Netherlands; 4 Department of Cell Biology, UMC Utrecht, Utrecht, The Netherlands

**Keywords:** extracellular vesicles, exosomes, RNA, proteomics, hypoxia, tumor necrosis factor alpha

## Abstract

**Background:**

The healthy vascular endothelium, which forms the barrier between blood and the surrounding tissues, is known to efficiently respond to stress signals like hypoxia and inflammation by adaptation of cellular physiology and the secretion of (soluble) growth factors and cytokines. Exosomes are potent mediators of intercellular communication. Their content consists of RNA and proteins from the cell of origin, and thus depends on the condition of these cells at the time of exosome biogenesis. It has been suggested that exosomes protect their target cells from cellular stress through the transfer of RNA and proteins. We hypothesized that endothelium-derived exosomes are involved in the endothelial response to cellular stress, and that exosome RNA and protein content reflect the effects of cellular stress induced by hypoxia, inflammation or hyperglycemia.

**Methods:**

We exposed cultured endothelial cells to different types of cellular stress (hypoxia, TNF-α-induced activation, high glucose and mannose concentrations) and compared mRNA and protein content of exosomes produced by these cells by microarray analysis and a quantitative proteomics approach.

**Results:**

We identified 1,354 proteins and 1,992 mRNAs in endothelial cell-derived exosomes. Several proteins and mRNAs showed altered abundances after exposure of their producing cells to cellular stress, which were confirmed by immunoblot or qPCR analysis.

**Conclusion:**

Our data show that hypoxia and endothelial activation are reflected in RNA and protein exosome composition, and that exposure to high sugar concentrations alters exosome protein composition only to a minor extend, and does not affect exosome RNA composition.

Exosomes are small, spherical vesicles that are secreted upon fusion of the limiting membrane of multivesicular bodies with the plasma membrane (1,2). They are produced by many cell types and are involved in intercellular signalling (3,4). As a result of inward budding during the formation of the intraluminal vesicles, exosomes contain cytosolic content, including proteins, mRNAs and miRNAs (5–7). Exosome-mediated signalling can occur through receptor-ligand interactions between membrane-bound proteins on the exosome and corresponding receptors on the cell surface (8). Exosome content can also be transferred into target cells, either through direct fusion of exosomes with the cell membrane or through active uptake, mediated by endocytosis (4,9,10). The latter 2 mechanisms would not only allow intercellular communication through protein interactions but through RNAs and cytoplasmic (signalling) proteins as well (6).

As mediators of intercellular communication, exosomes are involved in cellular stress responses. The secretion of stress-induced heat shock protein (Hsps) by several cells, including tumour cells, B lymphocytes, astrocytes and endothelial cells, has been described to be exosome associated (11–16). Exosomal Hsps are present on the surface of exosomes and can modulate immunological responses (16). Importantly, the presence of oxidative stress has been shown to alter RNA content of exosomes released by mouse mast cells, suggesting that stress-related signalling via exosomes does not only occur through protein transfer but also through shuttling of RNA (17).

The vascular endothelium, which forms the endothelial barrier between blood and the surrounding tissues, is known to efficiently respond to stress signals such as hypoxia and inflammation by adaptation of cellular physiology and secretion of (soluble) growth factors and cytokines that recruit endothelial progenitor cells or cells that play a role in the innate immune response (18). It has been demonstrated that endothelial cells also secrete exosomes (11,19) and several reports show that endothelial cells can also be targeted by exosomes derived from different cell types (6,20). Because of the important role of the vascular endothelium as a first line of defense against inflammatory, hypoxic and possibly also chronic stress (21–23), we hypothesized that endothelial cell-derived exosomes mediate communication of stress-related signals and that the content of these exosomes reflects the cellular stress in the cell of origin. We compared exosomes secreted by endothelial cells cultured under normal conditions, hypoxic conditions (model for ischemia), in the presence of TNF-α (model for inflammation and endothelial activation) and in the presence of high glucose (model for hyperglycemia) or mannose (osmotic control for glucose) concentrations. Exposure of endothelial cells to hypoxia or TNF-α modulated both protein and mRNA content of exosomes derived from these cells, whereas exposure to high glucose or mannose concentrations did not influence exosome protein or mRNA profiles. Our data indicate that protein and RNA content of endothelial cell-derived exosomes reflects signals of acute stress (hypoxic and endothelial activation) but not of short-term high sugar concentrations, present in the cells of origin.

## Materials and methods

### Cell culture

Human microvascular endothelial cells [HMEC-1; CDC; Atlanta, GA, USA (24)] were maintained (up to passage number 27) in MCDB131 medium (Life Technologies, Grand Island, NY, USA) containing 10% fetal bovine serum (FBS), 100 U/ml penicillin and 100 µg/ml streptomycin (all from Life Technologies, Grand Island, NY, USA), 50 nm Hydrocortisone, 10 mM L-glutamine and 10 ng/ml rhEGF at 37°C, 5% CO_2_ and ambient oxygen level (20%).

### Exosome isolation

Exosomes were isolated from the medium by differential centrifugation as described previously (25,26). Cells were grown to 70–75% confluence before exosome-free medium (prepared using FCS, which was centrifuged for at least 1 hour at 200,000*g* followed by 0.20 µm filter-sterilization) was added. During the 24-hour culturing period in exosome-free medium, cellular stress was induced by culturing in 2% O_2_ (hypoxia) or medium supplemented with 3.240 g/l glucose or mannose or 10 ng/ml TNF-α (all chemicals from Sigma, St Louis, MO, USA).

The culture medium was centrifuged sequentially for 15 min at 1,500*g*, 30 min at 10,000*g* and 60 min at 100,000*g* using a Beckman LE-80K centrifuge with SW32-Ti and SW60-Ti rotors (Beckman Instruments, Inc., Fullerton, CA, USA). Exosomes, pelleted in the 100,000*g* centrifugation step were washed twice by re-suspending in PBS and centrifugation at 100,000*g*.

### Quantitative proteomics

Exosomes were lysed in lysis buffer (2% SDS, 1% Triton-X100, 0.1 M Tris pH 7.4, 1 tablet Complete EDTA-free protease inhibitors (Roche, Indianapolis, IN, USA) and concentrations were determined using a BCA protein assay (Pierce, Rockford, IL, USA)). Samples from control and stress-exposed endothelial cells (100 µg each) were labelled with iTRAQ (isobaric tagging for relative and absolute quantitation) reagents (Life Technologies), purified and fractionated with strong cation exchange chromatography (SCX) and analysed on a Velos Orbitrap mass spectrometer. First, proteins were precipitated. Protein samples were mixed with 6 volumes of cold acetone (−20°C) and incubated for 60 min at −20°C. After 10 min of centrifugation at 13,000*g* at 4°C, the supernatant was removed and the rest of acetone was allowed to evaporate from the uncapped tubes at room temperature. Next, proteins were digested; for this, protein pellets were reconstituted with 20 µl of dissolution buffer (0.5 M triethylammonium bicarbonate), 1 µl denaturant (2% SDS) and 2 µl reducing reagent [50 mM tris-(2-carboxyethyl) phosphine]. The mixtures were mixed by vortexing, spun down and incubated at 60°C for 1 hour. Free cysteines were blocked by adding 1 µl of 200 mM Methyl methanethiosulfonate in isopropanol and incubated 10 min at room temperature. Trypsin (Promega V5111) was reconstituted with de-ionized water at 1 µg/µl concentration. Ten µl trypsin solution was added to each vial and incubated overnight at 37°C followed by iTRAQ labelling: 8-plex iTRAQ reagents were allowed to reach room temperature and then reconstituted with 50 µl of isopropanol. Each label reagent was mixed with the corresponding protein digest and incubated at room temperature for 2 hours. Samples were pooled into a new vial and dried using a centrifugal evaporator (Speedvac). After reconstituted with 0.1% formic acid (FA), the digest was desalted on an Oasis HLB 1 cc column (Waters, Milford, MA, USA) and eluted with 60% acetonitrile (ACN) 0.1% FA. Eluted peptide mixtures were dried by centrifugation evaporation, reconstituted with 100 µl SCX buffer A (10 mM KH_2_PO_4_, 20% ACN, pH 2.7) and separated on a PolyLC PolySULFOETHYL A column (200×2.1 m, 5 µm, 200 Å) with a linear 200 µl/min gradient of 0–70% buffer B (10 mM KH_2_PO_4_, 20% ACN, 500 mM KCl, pH 2.7) in 45 min on an Agilent 1200 LC device with Chemstation B.02.01 control software (Agilent, Santa Clara, CA, USA). Fractions were collected each minute and eventually pooled into 24 fractions. After vacuum centrifugation to evaporate the solute, fractions were desalted, eluted, dried as described above and reconstituted with 0.1% FA. Liquid chromatography was performed on an Eksigent nanoLC-Ultra 1D plus system (Eksigent, Dublin, CA, USA). Peptide digest was first loaded on a Zorbax 300SB-C18 trap (Agilent) at 6 µl/min for 5 min, then separated on a PicoFrit analytical column (100 mm long, ID 75 µm, tip ID 10 µm, packed with BetaBasic 5 µm 300 Å particles; New Objective, Woburn, MA, USA) using a 40-min linear gradient of 5–35% ACN in 0.1% FA at a flow rate of 250 nl/min. Mass analysis was carried out on an LTQ Orbitrap Velos (Thermo Fisher Scientific, San Jose, CA, USA) with data-dependent analysis mode, where MS1 scanned full MS mass range from m/z 300 to 2,000 at 30,000 mass resolution and 6 HCD MS2 scans were sequentially carried out at resolution of 7,500 with 45% collision energy, both in the Orbitrap.

### Database search and quantitative data analysis

MS/MS spectra from 24 fractions were searched against the Swiss Prot (Swiss Institute of Bioinformatics, updated August 10, 2010, 21,241 entries) database, taxonomy Human using our 6-processor Mascot (Matrix Science, London, UK; version 2.3) cluster at NIH (http://biospec.nih.gov), with precursor mass tolerance at 20 ppm, fragment ion mass tolerance at 0.05 Da, trypsin enzyme with 2 miscleavages, methyl methanethiosulfonate of cysteine and iTRAQ 8plex of lysine and the N-terminus as fixed modifications, and deamidation of asparagine and glutamine, oxidation of methionine and iTRAQ 8plex of tyrosine as variable modifications.

The resulting.dat file was load into Scaffold Q+ (version Scaffold_3_00_04, Proteome Software Inc., Portland, OR, USA) to filter and quantitate peptides and proteins. Peptide identifications were accepted at 95.0% or higher probability as specified by the Peptide Prophet algorithm (27) and a false discovery rate (FDR) of less than 1%. Protein identifications were accepted at 99.0% or higher probability and contained at least 2 identified peptides with FDR less than 1%. Protein probabilities were assigned by the Protein Prophet algorithm (28). Proteins that contained similar peptides and could not be differentiated based on MS/MS analysis alone were grouped to satisfy the principles of parsimony. Peptides were quantitated using the centroided reporter ion peak intensity, with minimum of 5% of the highest peak in the spectrum. Intrasample channels were normalised based on the median ratio for each channel across all proteins. The isobaric tagged samples were normalised by comparing the median protein ratios for the reference channel. Quantitative protein values were derived from only uniquely assigned peptides.

### Immunoblotting

Exosomes were lysed in lysis buffer, and concentrations were determined using a BCA protein assay (Pierce). Equal amounts of exosomes were subjected to 12% SDS-PAGE electrophoresis and transferred to PVDF membranes (Thermo Scientific). PVDF membranes were blocked in 5% low-fat dry milk powder (Campina, Amersfoort, The Netherlands) in TBS with 0.1% Tween-20 (blocking buffer). PVDF membranes were incubated with primary antibodies in blocking buffer, washed in TBS with 0.1% Tween-20 (TBST), followed by incubation with HRP-conjugated secondary antibodies in blocking buffer. Proteins were detected with Chemiluminescent Peroxidase Substrate (Sigma) and imaged on the Molecular Image ChemiDoc XRS system (Biorad, Hercules, CA, USA). After imaging, PVDF membranes were stripped from their antibodies using ReBlot Plus Mild Antibody Stripping Solution (Millipore, Bedford, NY, USA), blocked and re-probed for β-actin as a loading control. The primary antibodies used were β-actin (A5441, 1:15,000, Sigma), Flotillin-1 [sc-25506, 1:500, Santa Cruz Biotechnologies (Santa Cruz, CA, USA)], ICAM-I (SC-8439, 1:1,000, Santa Cruz Biotechnologies), LOXL2 [AF2639, 1:400, R&D Systems (Minneapolis, MN, USA)], PlexinA1 [PAB7879, 1:500, Abnova (Taipei City, Taiwan)], TNFAIP3 (MAB3716, 1:1000, Abnova), semenogelin-1 (SEMG1) [H00006406-B01P, 1:200, Novus Biologicals (Littleton, CO, USA)] and TUG (SC-101260, 1:200, Santa Cruz Biotechnologies). Secondary antibodies were HRP-conjugated swine anti-rabbit [P0399, 1:2,000, Dako (Glostrup, Denmark)], rabbit anti-Mouse (P0260, 1:2,000, Dako) and rabbit anti-goat (P0160, 1:2,000, Dako).

### Gene expression analysis

Exosomes were pelleted and washed twice with PBS. Total RNA was isolated using a mirVana RNA isolation kit (Invitrogen), concentration was determined using a Nanodrop spectrophotometer and RNA integrity was verified using Bioanalyzer pico RNA chips (Agilent). Labelled cRNA was generated according to the Illumina “whole-genome gene expression direct hybridization assay” protocol and, after determining the concentration using a NanoDrop spectrophotometer, hybridized on Illumina HumanHT12-v4 Beadchips (Illumina, San Diego, CA, USA). Gene expression data obtained from Illumina Beadstudio was normalised using “R” bioconductor with the “lumi” package (29). Genes that passed the detection call in >1 sample were included in the analysis. To identify differentially expressed genes between the various conditions, we created a linear model on the basis of moderated t-statistics using the “limma” package (30). Genes with a FDR <0.05 were considered significant.

### Quantitative reverse transcription-PCR (qRT-PCR)

cDNA was synthesized from same set of samples analysed by microarray analysis using the BioRad iScript cDNA Synthesis Kit. qRT-PCR analysis was performed on the BioRad CFX96 RT system, using the IQ Sybr Green Super Mix (Biorad). Primer sequences were taken from the Harvard Primer Databank (31) and synthesized by Sigma-Aldrich. The following primers were used: B2M (5′-CCAAGGAAGGCGTCTAAGGC-3′, 5′- TGCACTCCAGCAGTAGGTGT-3′), BNIP3 (5′-CAGGGCTCCTGGGTAGAACT-3′, 5′-CTACTCCGTCCAGACTCATGC-3′), CCL2 (5′-CAGCCAGATGCAATCAATGCC-3′, 5′-TGGAATCCTGAACCCACTTCT-3′), CIRBP (5′-AGGGCTGAGTTTTGACACCAA-3′, 5′-ACAAACCCAAATCCCCGAGAT-3′), GAPDH (5′-AAGGTGAAGGTCGGAGTCAAC-3′, 5′-GGGGTCATTGATGGCAACAATA-3′), IL-8 (5′-GAATGGGTTTGCTAGAATGTGATA-3′, 5′-CAGACTAGGGTTGCCAGATTTAAC-3′), IL-32 (5′-TGGCGGCTTATTATGAGGAGC-3′, 5′-CTCGGCACCGTAATCCATCTC-3′), NDRG1 (5′-TCGAGACTTTACATGGCTCTGT-3′, 5′-TCATGCCGATGTCATGGTAGG-3′), RPLP0 (5′-TCGACAATGGCAGCATCTAC-3′, 5′-ATCCGTCTCCACAGACAAGG-3′),SOD2 (5′-GCTCCGGTTTTGGGGTATCTG-3′, 5′-GCGTTGATGTGAGGTTCCAG-3′), TNIP1 (5′-CTAGTGTGACGGCAGGTAAGG-3′, 5′-GCTGCTTCATGGACCGGAA-3′) and VCAM (5′-GGGAAGATGGTCGTGATCCTT-3′, 5′-TCTGGGGTGGTCTCGATTTTA-3′). Ct values were normalised per gene and experiment. ddCt was calculated using the geometric mean of 3 common housekeeping genes, which did not show any variation in the genomic screening (RPLP0, B2M, and GAPDH). Statistical analysis was performed by 2-tail Student t-test. Differences were considered statistically significant at p < 0.05.

### Gene ontology and pathway enrichment analysis

Gene Ontology Term for Term analysis was performed using Ontologizer (32,33). The p-values for significant overrepresentation of gene ontology terms in the identified gene set as compared to human genes was calculated using a modified Fisher exact test (1-tailed) to which a Benjamini-Hochberg correction for multiple testing was applied with the significance threshold at p = 0.0100.

### Nanoparticle tracking analysis (NTA)

Size distribution of vesicles in 100,000*g* pellets were analysed using a Nanosight LM10-HS (NanoSight, Amesbury, UK) with a 532-nm laser-equipped sample chamber. The sample chamber was completely filled with 100,000*g* pellets resuspended in PBS. Shutter and gain were manually adjusted for optimal detection and were kept at this setting for all samples. A 1-min AVI file was recorded and analysed using NTA (version 2.2, build 0370, Nanosight) software to calculate size distributions and vesicle concentrations using the following settings: calibration: 166 nm/pixel; blur: auto; detection threshold: 14, minimum track length: auto, temperature: 22°C, viscosity: 0.95 cP. Differences in vesicle concentration and modal sizes were analysed by 1-way ANOVA using a Bonferroni-Holm post-hoc test to correct for multiple testing, assuming p-values <0.05 to be significant.

### Electron microscopy

Transmission electron microscopy was performed as described by Slot and Geuze (34). Briefly, carbon-coated Formvar filmed grids were placed on exosome suspension for 20 min and washed with 0.15% glycine in PBS 3 times followed by a 0.1% BSA in PBS wash. Vesicles were fixed in 1% glutaraldehyde in PBS for 5 min and washed twice with PBS. For CD63 labelling, grids were placed on 5 µl 1% BSA in PBS containing 5 µg/ml anti-human CD63 (CLB, Amsterdam, The Netherlands) for 20 min, washed 4 times with 0.1% BSA in PBS and incubated on a drop of 1% BSA in PBS containing rabbit anti-mouse polyclonal antibody (Dako) for 20 min. Secondary antibodies were labelled by incubation on PBS containing 1% BSA and 10 nm gold particles coupled to protein A for 10 min. Specimen were fixed in 1% glutaraldehyde in PBS for 5 min and washed twice with PBS. After 4 washes on distilled water, grids were placed on ice-cold 1.8% methylcellulose (25 Ctp)/0.4% uranyl acetate (MC-AU) for 5 min, and after drying, vesicles were visualised using a FEI Tecnai 12 (FEI, Hillsboro, OR, USA) transmission electron microscope.

### Sucrose gradient analysis

The 100,000*g* pellets were resuspended in 250 µl 2.5 M sucrose, 20 mM TRIS HCl pH 7.4 and floated in a SW60 tube for 16 hours at 190,000*g* using a linear sucrose gradient (2.0–0.25 M sucrose, 20 mM Tris-HCl, pH 7.4). Gradient fractions (250 µl) were collected from the top used for subsequent immunoblot and electron microscopy analyses.

## Results

### Sample characterization

Cultured endothelial cells produce exosomes and microvesicles, which can be isolated from the culture medium (11,19,35,36). Vesicles present in the obtained 100,000*g* pellet (after 2 PBS washes) of cells grown under standard condition (no stress) were characterized with respect to their density, shape and size using sucrose density centrifugation, transmission electron microscopy and NTA.

**Fig. 1:**
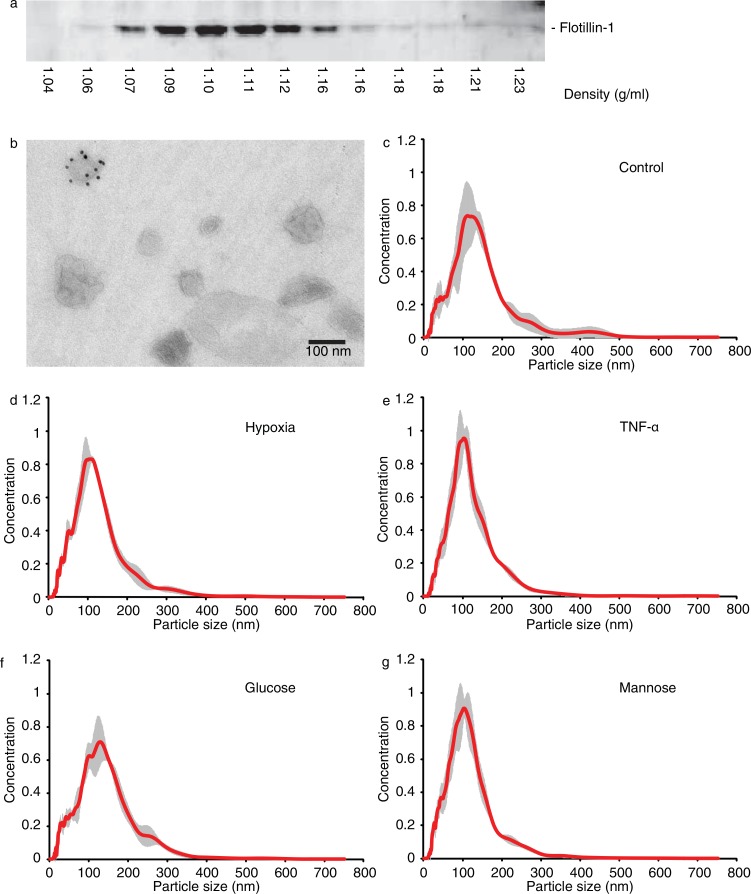
Characterization of endothelial cell-derived exosomes. Vesicles in the final, washed 100,000*g* pellets from endothelial cell culture supernatant cultured under standard conditions were analysed by sucrose density gradient and subsequent immunoblotting for Flotillin-1 (a), transmission electron microscopy (b; 10 nm gold: CD63) and by NTA (c). Size distributions of vesicles in washed 100,000*g* pellets from supernatants of stress-exposed endothelial cells were compared by NTA (d–g; conditions indicated, grey area represents SD).

Immunoblotting of sucrose density gradient fractions for Flotillin-1 reveals bands in fractions between 1.07 and 1.16 g/ml with peak fractions at a density of 1.10–1.11 g/ml (Fig. 1a). Transmission electron microscopy analysis of vesicles in the pooled 4 peak fractions (1.09–1.12 g/ml) shows vesicles at sizes around 100 nm, of which some contain CD63 protein. Also, larger vesicles could be detected (Fig. 1b), and NTA of the 100,000*g* pellet (Fig. 1c) confirmed the observed size distribution. Based on isolation procedure and vesicle characteristics, we term the vesicles present in the 100,000*g* pellet, which were used for subsequent analyses, exosomes, although we accept that our preparation also contains larger microvesicles.

Subsequently, endothelial cells were grown under different stress conditions (hypoxia, TNF-α or high glucose) in exosome-depleted medium for 24 hours. Cells were grown in high mannose medium to serve as an osmotic control, allowing us to distinguish effects of high glucose from changes induced by osmotic stress. Washed 100,000*g* pellets were analysed by Nanosight analysis to investigate effects of cellular stress on vesicle size distribution, revealing that cellular stress did not significantly affect vesicle size or concentration (Fig. 1d–g; supplemental information Table SI).

### Protein content confirms cellular origin of exosomes

To investigate whether the stress conditions under which our endothelial cells were cultured are reflected in exosome protein content, we performed iTRAQ-based quantitative proteomics analysis (37,38). Exosomal proteins were digested, labelled and analysed using LC-MS/MS. Accepting protein identifications based on identification of at least 2 peptides with a probability higher than 95% and minimal total protein identification probability of 99% resulted in the identification of 1,354 proteins (Supplemental Information, Table SII). Analysis of enriched gene ontology (GO)-terms (32) representing cellular component classes (Supplemental Information, Fig. S1) reveals that proteins annotated to cytoplasmic content were abundantly present in the isolated exosomes (GO-terms “intracellular,” “cytosol,” “ribosome” and “ribonuclear complex”). Furthermore, GO-terms indicating that cellular vesicles are analysed (“vesicle,” “membrane-bounded vesicle” and “cytoplasmic membrane-bounded vesicle”) are significantly enriched. Clues on the origin of the protein sample are provided by the significantly enriched GO-terms “melanosome,” “pigment granule,” “endosomal part” and “endosome membrane,” reflecting lysosome-related organelles that have previously been identified as enriched in exosome protein samples (39).

### Cellular stress conditions are reflected in exosome protein levels

To compare the proteome of exosomes from cells grown under different conditions, all iTRAQ-labelled peptides were quantified. Two identical (control condition) exosome samples were included to assess reproducibility of quantitation between samples. All proteins for which the Log^2^ ratio of these 2 samples was between −0.2 and 0.2 and for which at least 2 peptides could be reliably quantified were further analysed (1,240 proteins, Supplemental Information, Table SIII). After manual inspection of quantified peptides, differentially abundant proteins could be identified for all stress conditions. Exposure to hypoxic conditions or TNF-α yielded several up- or downregulated proteins, whereas high glucose and mannose concentrations affected the abundance of only few proteins (Table I and Supplemental Information, Table SIII).

**Table I:** Proteins showing differential abundances: top up- and downregulated proteins, compared to control (>1.25-fold)

Condition	Protein	Full name	NP number	Fold (±SD)
Hypoxia	SEMG1	Semenogelin 1	NP_002998.1	6.39 (2.44)
	CO4A	Complement component 4A	NP_009224.2	2.56 (0.77)
	LOXL2	Lysyl oxidase-like 2	NP_002309.1	2.30 (0.53)
	CO1A1	Collagen, type 1, alpha 1	NP_000079.2	0.74 (0.27)
	AN32E	Acidic (leucine-rich) nuclear phosphoprotein 32 family, member E	NP_112182.1	0.79 (0.24)
	EPN1	Epsin 1	NP_037465.2	0.84 (0.23)
TNF-α	TNIP1	TNFAIP3 interacting protein 1	NP_006049.3	6.60 (4.70)
	TNFAIP3	Tumor necrosis factor, alpha-induced protein 3	NP_006281.1	6.60 (2.50)
	ICAM1	Intracellular adhesion molecule 1	NP_000192.2	1.93 (0.41)
	CO5	Complement component 5	NP_001726.2	0.79 (0.09)
	APOM	Apolipoprotein M	NP_061974.2	0.84 (0.22)
	COIA1	Collagen, type XVIII, alpha 1	NP_569712.2	0.88 (0.18)
Glucose	ASPC1	Alveolar soft part sarcoma chromosome region, candidate 1	NP_076988.1	1.49 (0.80)
	TENX	Tenascin-XB	NP_115859.2	1.27 (0.39)
	CO4A	Complement component 4A	NP_009224.2	0.47 (0.17)
	CO5	Complement component 5	NP_001726.2	0.77 (0.13)
Mannose	SMD3	Small nuclear ribonucleoprotein D3 polypeptide 18 kDa	NP_004166.1	1.64 (0.26)
	SFXN1	Sideroflexin 1	NP_073591.2	1.36 (0.25)
	CO4A	Complement component 4A	NP_009224.2	0.48 (0.14)

**Fig. 2:**
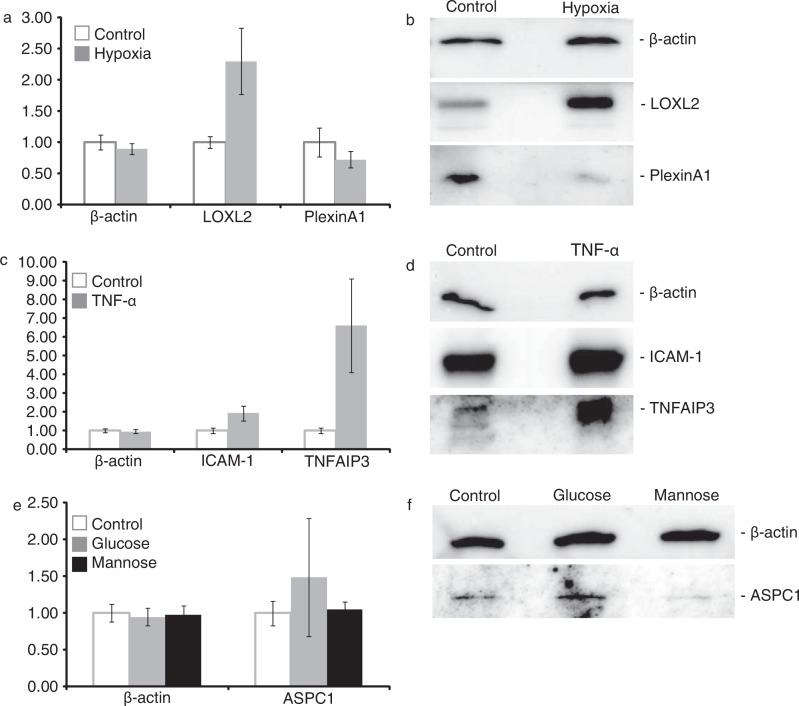
Verification of quantitative proteomics data by immunoblotting. Differences observed in quantitative proteomics, expressed as mean±SD based on analysis of individual quantified peptides (a–c) were verified by immunoblotting (d–f).

To confirm the quantitative proteomics data, differential abundances of a subset of proteins (based on availability of usable antibodies) were investigated using immunoblot analysis. β-Actin, a protein that was not affected in any condition, was used as a loading control. In agreement with the quantitative proteomics data (Fig. 2a), a decrease in Plexin A1 and an increase in lysyl oxidase like-2 (LOXL2) were observed in exosomes from endothelial cells cultured under hypoxic conditions (Fig. 2b). The increase of intercellular adhesion molecule 1 (ICAM-1) and tumor necrosis factor, alpha-induced protein 3 (TNFAIP3) in exosomes from endothelial cells cultured with 10 ng/ml TNF-α could also be confirmed (Fig. 2c,d). A minor increase of alveolar soft part sarcoma chromosomal region candidate gene 1 (ASPC1) was observed in exosomes from cells cultured in high glucose medium, but not in high mannose medium, in line with the quantitative proteomics analysis (Fig. 2e,f).

### Gene expression analysis reveals stress-related exosomal mRNA abundances

**Fig. 3:**
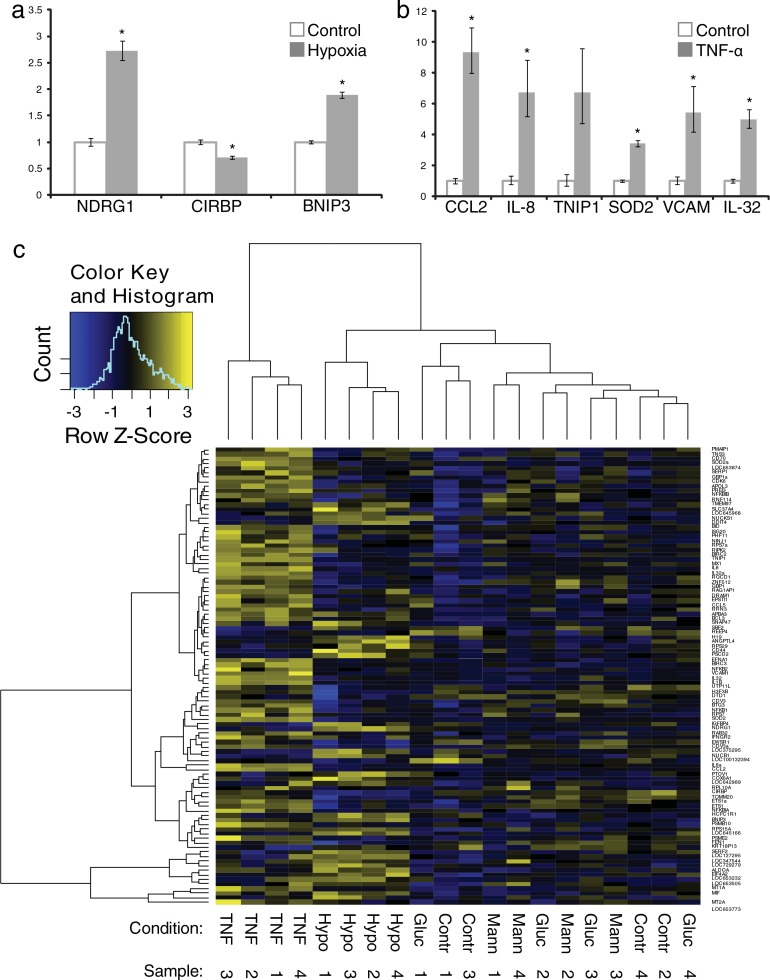
Analysis and verification of exosome mRNA profiles. Quantitative PCR confirms differential abundance of mRNAs identified to be significantly increased or decreased in exosomes from hypoxia (a) or TNF-α exposed cells compared to those from control cells (b), error bars represent SEM, *p < 0.05. Clustering analysis based on the 100 most variable mRNAs distinguishes exosomes from endothelial cells exposed to hypoxia or TNF-α, but not to high glucose or mannose, from control cell-derived exosomes (c).

To determine whether mRNA profiles in exosomes from stress-exposed endothelial cells vary between conditions, total RNA was isolated from washed 100,000*g* pellets followed by gene expression analysis on Illumina bead chip microarrays. After background subtraction, 10,533 mRNAs were confirmed to be present in endothelial cell-derived exosomes in more than 1 out of 24 samples, and 1,992 mRNAs were detected in all samples (Supplemental Information, Table SIV). We identified 18 genes that were significantly up- or downregulated in exosomes isolated from TNF-α treated cells and 3 genes in exosomes from cells cultured under hypoxia. No genes were significantly up- or downregulated in high-glucose conditions or in the isomolar mannose control (Table II). We used RT-qPCR to verify significant differentially abundant RNAs observed in hypoxia (Fig. 3a) and 6 of the significantly upregulated mRNAs in exosomes from TNF-α treated cells (Fig. 3b) and confirmed differential abundance of all these mRNAs except for that encoding TNFAIP3-interacting protein 1 (TNIP1).

**Table II:** RNAs showing significant differential abundances (hypoxia, TNF-α): differentially abundant mRNAs

Condition	Gene	NM number	Fold change	Adj. p value
Hypoxia	NDRG1	NM_006096.2	1.376	0.003
	CIRBP	NM_001280.1	0.810	0.029
	BNIP3	NM_004052.2	1.231	0.044
TNF	CCL2	NM_002982.3	3.242	0.000
	IL8	NM_000584.2	4.103	0.000
	TNIP1	NM_006058.3	1.500	0.000
	IL1B	NM_000576.2	1.396	0.000
	SOD2	NM_001024465.1	1.701	0.000
	VCAM1	NM_001078.2	1.251	0.000
	IL32	NM_001012633.1	1.706	0.000
	BIRC3	NM_001165.3	1.221	0.000
	NFKB1	NM_003998.2	1.358	0.003
	EFNA1	NM_004428.2	1.205	0.004
	CCL5	NM_002985.2	1.218	0.011
	RPS7	NM_001011.3	1.285	0.011
	BIRC2	NM_001166.3	1.338	0.017
	APBA3	NM_004886.3	1.121	0.025
	MT1A	NM_005946.2	1.815	0.027
	CDV3	NM_017548.3	1.241	0.032
	NFKBIA	NM_020529.1	1.648	0.045
	LOC375295	XM_374020.4	0.835	0.046

Using unsupervised hierarchical clustering of the top 100 genes that showed the highest intergroup variance (based on moderated t-statistics), we observed that mRNA profiles from exosomes isolated from TNF-α- and hypoxia-exposed cells consistently clustered together, whereas profiles of exosomes from glucose- and mannose-treated cells could not be distinguished from untreated controls (Fig. 3c).

## Discussion

The data presented here demonstrate that protein and RNA levels in exosomes secreted by endothelial cells varies depending on culture condition and type of stress to which the cells are exposed to, suggesting that endothelial cells employ exosome-mediated cell communication to cope with conditions of cellular stress. Using sucrose gradient analysis and transmission electron microscopy, the isolated vesicles are demonstrated to contain characteristics that are typical for exosomes. Although some contamination with larger vesicles could be detected, the size distribution appears comparable to exosomes isolated from human umbilical vein endothelial cell (HUVEC)-derived exosomes (19). We subjected exosomal proteins and mRNAs to quantitative proteomics and gene expression analyses, identifying 1,354 proteins and 1,992 mRNAs, respectively. Analysis of enriched GO-terms provided confidence that our sample contained membrane-bounded vesicles with characteristics of endosomal vesicles and lysosome-related organelles, similar to the characteristics of B cell-derived exosomes (39). Furthermore, in agreement with prior exosome proteomics studies, components of the ESCRT complex and exosomal marker proteins as Flotillin-1 and -2, CD9, CD63, heat shock protein 70 (HSP70), MHC molecules, CD81, CD82 and proteins from the Rab family could be identified in our samples (3,7,39,40). The tetraspanin protein CD63 was demonstrated to be present on a subset of exosomes, in line with the findings by van Niel et al. (41).

Nanoparticle tracking analysis was used to investigate whether cellular stress affects exosome size and concentration, and although minor differences could be detected, these differences did not appear significant. Using quantitative approaches to analyse exosomal protein and mRNA content, we demonstrate that exosomes from cells exposed to hypoxia or TNF-α could be clearly distinguished from control exosomes (and each other). Exposure to high glucose or mannose (osmotic control) concentrations resulted in minor differences in protein profiles and no significantly altered mRNA levels.

We verified the quantitative proteomics data by immunoblotting, and although changes in abundance were confirmed for proteins representing all stress conditions, some puzzling results were obtained. SEMG1, upregulated in exosomes from hypoxic cells, showed different band patterns in whole cell lysates and exosome samples (Supplemental Information, Fig. S2a). The lower band, detected in exosome sample, may represent a product of SEMG1 cleavage (42,43), suggesting that this product is enriched in exosomes from endothelial cells grown in hypoxic conditions. The TNF-α induced increase in TNFAIP3 was confirmed by immunoblot analysis. However, the observed signal corresponds to a protein (-complex) of >170 kDa instead of the predicted size of 90 kDa (Supplemental Information, Fig. S2b), potentially reflecting protein aggregates containing TNFAIP3 due to the non-denaturing conditions used in this analysis.

We used RT-qPCR to validate a selection of differentially abundant mRNAs. Since no common housekeeping genes for RT-qPCR in exosomes are available, the relative changes were determined by comparing mRNA expression of several affected genes with the geometric mean of 3 commonly used housekeeping genes, which were unaffected by all conditions in the gene expression analysis. The magnitudes of changes differed from the changes observed in the gene expression analysis but all changes investigated in this manner could be verified, confirming that indeed exosomal RNA levels reflect cellular stress.

Treatment of HMEC-1 cells with high concentrations of glucose or mannose affected only few proteins and had no significant effect on any of the mRNAs found in exosomes. Potentially, our experimental setup does not induce sufficient stress to be reflected in exosomes from these cell, and exposure time (44) and sugar concentrations may need to be increased. A minor increase of ASPC1 was observed in exosomes from endothelial cells cultured in high glucose medium but not for a similar concentration of mannose (in line with the quantitative proteomics analysis). In contrast, complement component 4A (CO4A) is equally downregulated in exosomes from cells exposed to glucose and mannose, indicating an effect of osmotic stress.

The major changes observed in exosomes from TNF-α-exposed cells involved proteins that are commonly affected by TNF-α in endothelial cells, such as ICAM-1 and TNFAIP3 (45–47), and interestingly, our data are consistent with the observed upregulation of ICAM-1 in endothelial microparticles from TNF-α-treated endothelial cells in a qualitative proteomics study (48). Our gene expression analysis revealed 18 genes significantly up- or downregulated in exosomes from TNF-α treated cells. The genes and proteins affected by TNF-α display a variety of functions, including superoxide protection (superoxide dismutase 2, SOD2), immune responses (chemokine (C-C) ligand 2, interleukin-8 and -32, vascular adhesion molecule 1) and the NF-κB pathway (NF-kappa B, NF-kappa BIA, TNIP1). These changes represent the typical protein and gene expression profiles of activated endothelial cells (49–54), clearly reflecting the stress condition of exosome producing cells.

Proteins found upregulated in exosomes from cells grown under hypoxia suggest that cytoskeletal and extracellular matrix rearrangements can be induced by these exosomes, involving proteins such as LOXL2, fibronectin and collagen. Induction of such processes by hypoxia in endothelial cells is common (55), and because of their secretion into the environment, exosomes may especially contribute to extracellular matrix remodelling. In contrast, changes identified in the microarray analysis concern genes involved in stress response (N-myc downstream regulated 1, cold inducible RNA binding protein) and apoptosis (BCL2/adenovirus E1B 19 kDa interacting protein 3) (56–59). Taken together, both protein and mRNA content of exosomes from endothelial cells cultured under hypoxic conditions are affected and distinguish them from control cell-derived exosomes.

Although it remains to be determined whether exosomes from stressed endothelial cells also serve different functional goals, our data underscore that the content of exosomes can be used to assess the physiological condition of their producing cells. This principle has increased the interest in exosomes as a source for disease biomarkers, for example, for tumours and kidney diseases (60–64).

Altogether, our study demonstrates that cellular stress conditions are reflected in exosome protein and RNA content and suggest a role for endothelial exosomes in the transfer of stress signals to target cells in case of stress, activation and damage, as well as disease.
